# Isolation and characterization of a novel mycobacteriophage Kashi-VT1 infecting *Mycobacterium* species

**DOI:** 10.3389/fcimb.2023.1173894

**Published:** 2023-07-21

**Authors:** Tanmayee Nayak, Anuja Kakkar, Rakesh Kumar Singh, Lav Kumar Jaiswal, Anand Kumar Singh, Louise Temple, Ankush Gupta

**Affiliations:** ^1^ Molecular Microbiology Laboratory, Department of Biochemistry, Institute of Science, Banaras Hindu University, Varanasi, India; ^2^ Interdisciplinary School of Life Sciences, Institute of Science, Banaras Hindu University, Varanasi, India; ^3^ School of Integrated Sciences, James Madison University, Harrisonburg, VA, United States

**Keywords:** mycobacteriophage, phage therapy, KVT1, latency period, burst size, antibiotic resistance

## Abstract

Mycobacteriophages are viruses that infect members of genus *Mycobacterium*. Because of the rise in antibiotic resistance in mycobacterial diseases such as tuberculosis, mycobacteriophages have received renewed attention as alternative therapeutic agents. Mycobacteriophages are highly diverse, and, on the basis of their genome sequences, they are grouped into 30 clusters and 10 singletons. In this article, we have described the isolation and characterization of a novel mycobacteriophage Kashi-VT1 (KVT1) infecting *Mycobacterium >smegmatis* mc^2^ 155 (*M. smegmatis*) and *Mycobacterium fortuitum* isolated from Varanasi, India. KVT1 is a cluster K1 temperate phage that belongs to *Siphoviridae* family as visualized in transmission electron microscopy. The phage genome is 61,010 base pairs with 66.5% Guanine/Cytosine (GC) content, encoding 101 putative open reading frames. The KVT1 genome encodes an immunity repressor, a tyrosine integrase, and an excise protein, which are the characteristics of temperate phages. It also contains genes encoding holin, lysin A, and lysin B involved in host cell lysis. The one-step growth curve demonstrated that KVT1 has a latency time of 90 min and an average burst size of 101 phage particles per infected cell. It can withstand a temperature of up to 45°C and has a maximum viability between pH 8 and 9. Some mycobacteriophages from cluster K are known to infect the pathogenic *Mycobacterium tuberculosis* (*M. tuberculosis*); hence, KVT1 holds potential for the phage therapy against tuberculosis, and it can also be engineered to convert into an exclusively lytic phage.

## Introduction

Tuberculosis (TB) is a chronic communicable disease that mostly affects not only the lungs (pulmonary TB) in the form of tubercular infections but also other organs (extrapulmonary TB) like the brain, kidneys, and spine in the form of non-tubercular *Mycobacterium* infections. It can be in an active form or can remain latent for a lifetime (Fisher and Elwood, 2013).

TB is one among the three major infectious diseases along with HIV/AIDS and COronaVIrus Disease of 2019 (COVID-19), which results in highest deaths worldwide. In the year 2021, 1.6 million individuals worldwide lost their lives to TB including 187,000 people with HIV. TB is the second most common infectious killer in the world, behind COVID-19 and above HIV/AIDS, and is the 13th highest cause of death overall (World Health Organization, 2022; Furin et al., 2019). Robert Koch first identified *M. tuberculosis* as the causative agent of human TB more than a century ago, but, even today, it still poses a major threat to human health (Cambau and Drancourt, 2014; Maitra et al., 2019). *M. tuberculosis* is very effective as a pathogen owing to its resistance to most well-known antibiotics, and it can precisely detect host immune reactions to make appropriate adjustments to their life cycle (Koul et al., 2011; Torfs et al., 2019). From the early 1990s until date, antibiotics are the mainly available treatment for *M. tuberculosis* infections and are administered for prolonged durations (normally, 6–9 months) in patients with TB.

Development of drug resistance in *M. tuberculosis* is one of the major challenges in its treatment, which mainly arises due to monotherapy, insufficient doses of antibiotics, treatment obstruction, and drug interactions (Moreno-Gamez et al., 2015; Khawbung et al., 2021). Drugs alone will not ultimately be able to treat TB due to frequent mutations in the pathogen that results in drug resistance. Alternative treatment options include probiotics, nanobiotics, antibody-antibiotic conjugates, vaccines, stem cell–based small inhibitory peptides, bacteriocins, small interfering RNA (siRNAs), silver nanoparticles, clustered regularly interspaced short palindromic repeats (CRISPR)–Cas editing machinery, and bacteriophage (phage) therapy (Laurenzi et al., 2007; Zumla et al., 2015; Wei et al., 2020). In the current scenario, antimicrobial resistance to standard TB treatments is rampant. The majority of current therapies target the *M. tuberculosis* cell envelope, a complex structure primarily made of lipids and carbohydrates, but it is difficult to create new and efficient medications because little is known about the structure of emerging drug-resistant *M. tuberculosis* cell envelopes and how they adapt to the pulmonary environment (Garcia-Vilanova et al., 2019). Because mycobacteriophages are natural antagonists of mycobacteria that amplify several folds in the presence and become undetectable in the absence of host, phage therapy can be considered an important alternative to antibiotics in the treatment of drug-sensitive, multidrug-resistant (MDR), and extensively drug-resistant (XDR) mycobacterial infections. Although *M. tuberculosis* is an intracellular pathogen residing within the macrophages, a study by (Broxmeyer et al., 2002) describes a system using *M. smegmatis*, an avirulent *Mycobacterium*, to deliver the lytic phage TM4 into the macrophages hosting both *M. avium* and *M. tuberculosis* with a marked reduction in the number of viable intracellular bacilli. Phages not only are the most decent bactericidal agents but also have narrower potential for inducing resistance, have low environmental impact, are equally effective against both antibiotic-sensitive and antibiotic-resistant bacteria, are able to replicate unlike that of antibiotics, and cause a minimal disruption of the normal human flora.

In our study, we have focused on phage therapy as one of the alternatives of antibiotics. In phage therapy, bacteriophages (phages) are used to treat bacterial infections (Housby and Mann, 2009). Phages are viruses that infect bacterial hosts and are known to be the most abundant entities in the biosphere that requires their specific bacterial host for replication and propagation (d'Herelle and Smith, 1926). The therapeutic strategy first appeared at the beginning of the 20th century, but, following the Second World War, the antibiotic use gradually became prevalent in the majority of the world. During the 1980s, Western scientists “rediscovered” phage therapy as a response to the growing risk of emerging antibiotic resistant strains (Carlton, 1999). Mycobacteriophages are phages that infect the genus *Mycobacterium*, and all mycobacteriophages discovered to date are double-stranded DNA (dsDNA) tailed ones that morphologically fall under either *Siphoviridae* or *Myoviridae* family based on their structure and appearance. Most mycobacteriophages were isolated using the host organism *M. smegmatis* (Hatfull, 2019), which is a fast-growing member of genus *Mycobacterium*. *M. smegmatis*, which is least/non-pathogenic as compared to *M. tuberculosis*, has been substituted for the extremely pathogenic *M. tuberculosis* in many molecular biology or molecular genetic experiments. Characteristics such as non-pathogenicity, rapid growth, and similarities with other mycobacteria make *M. smegmatis* a suitable laboratory strain for various experiments related to mycobacteria and for phage isolations (Gordon and Smith, 1953; Ranjitha et al., 2020). When comparing orthologous pairs between pathogenic and non-pathogenic species to determine whether it is appropriate to use them as substitute systems for pathogenic species, orthologs of four pathogenic species—*M. ulcerans*, *M. tuberculosis*, *M. leprae*, and *M. marinum*—found in the genomes of *M. smegmatis* were shown to have an average identity of over 70% (Malhotra et al., 2017). There is even a greater identity of the genetic backgrounds between *M. smegmatis* and *M. tuberculosis*; hence, it is a promising candidate for initial phage screenings. Several polyvalent phages have been already isolated that were screened with *M. smegmatis* and infect both *M. smegmatis* and *M. tuberculosis* (Jacobs-Sera et al., 2012).

The isolation and genomic characterization of mycobacteriophages have shed light on viral diversity and evolution in addition to the creation of tools for mycobacterial genetics. To date, more than 12,000 mycobacteriophages have been isolated out of which more than 2,000 mycobacteriophages are sequenced (PhagesDB.org). Mycobacteriophages are genetically very diverse and are categorized into 30 different types of clusters and at least 10 singletons thus far (Jacobs-Sera et al., 2012).

Understanding and treating human mycobacterial infections may benefit greatly from the study of phages with mycobacterial hosts. A large repertoire of diverse and fully characterized mycobacteriophages can be a very useful asset in the fight against drug-resistant *M. tuberculosis* infections. A single phage or cocktails of wild-type and engineered mycobacteriophages are currently being utilized in the phage treatment to treat disseminated drug-resistant cutaneous *M. abscessus* and *M. chelonae* infections (Dedrick et al, 2019; Little et al., 2022). A method has also been devised to test the antibiotic resistance of *M. tuberculosis* strains using mycobacteriophages containing a reporter gene (Sarkis et al., 1995). These utilities and applications of mycobacteriophages led us to isolate and characterize the mycobacteriophage Kashi-VT1 (KVT1).

## Materials and methods

### Host strain and culture conditions

For isolation and characterization of novel mycobacteriophage, *Mycobacterium smegmatis* mc^2^ 155 (*M. smegmatis*), ATCC700084 strain was used as the host organism. The host cells were grown on Luria Bertani (LB) Agar (2% w/v) plates supplemented with 0.4% (v/v) glycerol and 0.1% (v/v) Tween 80 (LBGT media) at 37°C for 48 h at 150 rotations per minute (rpm) in a rotary shaker (Sun et al., 2018). For primary liquid culture, cells were grown in LBGT media at 37°C in a rotary shaker. For isolation, propagation, and characterization of mycobacteriophage, host cells were grown up to mid-log phase [~0.5 Optical Density (OD)] in LBGT media. To form bacterial lawn on double-layer agar plates, bottom agar was prepared with 2% w/v LBG agar. Top agar consisting of 0.8% w/v LBG agar was mixed with 0.5 ml of host culture and poured onto the bottom agar followed by incubation at 37°C.

### Isolation and propagation of bacteriophage

A total of 48 samples were collected from different locations inside the campus of Banaras Hindu University, Varanasi, India. Isolation of mycobacteriophage was carried out as described in PhagesDB database with minor modifications (Sarkis and Hatfull, 1998). The samples collected for mycobacteriophage isolation were moist soil samples. Collected soil sample (~2–3 g) was mixed with 3 ml of phage buffer [10 mM Tris-HCl (pH 7.5), 10 mM MgSO_4_, 68.5 mM NaCl, and 1 mM CaCl_2_] and incubated in a rotary shaker at 37°C for 150 rpm for 1 h. The supernatant was collected after centrifugation at 10,000 rpm for 5 min and filtered through 0.22-µm filter paper. *M. smegmatis* culture was centrifuged at 5,000 rpm for 5 min, and the supernatant was discarded to remove Tween 80. Cells were again resuspended in LBG liquid medium. The sample filtrate (0.1 ml) was mixed with 0.5 ml of log phase *M. smegmatis* culture in LBG medium and incubated at 37°C for 30 min followed by plating on to double-layer agar plates. Again, the plates were incubated at 37°C for 48 h and examined for appearance of plaque.

### Naming of novel mycobacteriophage

KVT1 was named for Kashi, the ancient name of Varanasi city, and Vishwanath Temple area inside the campus of Banaras Hindu University where it was isolated.

### Host range determination

In this experiment, apart from the host *M. smegmatis*, specificity of KVT1 was also tested against *M. fortuitum* and bacterial strains other than *Mycobacterium* species, *viz*., *Escherichia coli* (DH5α and BL21 strains), *Pseudomonas aeruginosa* (ATCC 27553), *Enterococcus faecalis* (ATCC 29212), *Staphylococcus aureus* (ATCC 2542), and *Klebsiella pneumoniae* [American Type Culture Collection (ATCC) 1705]. For this experiment, briefly, 0.45 ml of pre-heated LBG agar (0.8%) was combined with 0.5 ml of each bacterial strain along with *M. smegmatis* as control and was uniformly poured onto LBG bottom agar. Phage KVT1 at a concentration of 10^9^ plaque-forming units (PFU)/ml was applied to the surface of each plate in an amount of around 0.01 ml. The plates were incubated at 37°C for 48 h. By observing the presence or absence of lytic zones on the plates, the host range is ascertained.

### Transmission electron microscopy

Morphology of mycobacteriophage KVT1 was visualized using a transmission electron microscope (TEM) (Ackermann, 2009). A drop of bacteriophage at a concentration of 10^10^ Plaque forming unit (PFU)/ml was applied onto the carbon-coated copper grid and incubated for 1 min. The grid was rinsed with sterile water and stained with 1% phosphotungstic acid (PTA) followed by incubation for 1 min and dried after removing excess liquid from the grid. Morphology of the phage was visualized using TECNAI 200 KV TEM (SAIF Facility, AIIMS, New Delhi).

### Genomic DNA isolationand restriction digestion of KVT1 genome

Mycobacteriophage KVT1 was plated onto double-layer LBG agar plates to form densely packed plaques. Phage buffer (5 ml) was poured onto the plates and incubated overnight. The next day, buffer from the plate was passed through 0.22-µm syringe filter. Phage DNA was extracted through phenol-chloroform-isoamyl (PCI)–Sodium dodecyl sulfate (SDS) method described in PhagesDB database (Nishiguchi et al., 2002). Briefly, the phage solution was incubated with Deoxyribonuclease (DNase) (0.1 U/ml) and Ribonuclease (RNase) A (4µg/ml), followed by treatment with Ethylene diamine tetra acetic acid (EDTA), SDS, and proteinase K followed by the PCI alcohol (25:24:1) DNA extraction method.

Mycobacteriophage DNA (100 ng/µl) was digested using *Pst* I, *Hind* III, and *Bam* HI and run on to 1% agarose gel. DNA fragments were visualized and counted. The use of restriction enzymes *Pst* I, *Hind* III, and *Bam* HI was decided as per instructions given in PhagesDB.com and seaphages.org. When digested, the variable numbers and/or lengths of DNA fragments result from differences in the number and/or locations of these restriction sites in the genomes of phages. This restriction pattern can be used as a fingerprint for a particular phage genome to compare with that of other phages especially in case of *Pst* I as it is abundant in the mycobacteriophage genome (seaphages.org) (Fleming et al., 2004).

### Genomic DNA sequencing and annotation

The whole genome sequencing of KVT1 was carried out by commercial vendor MedGenome, Bengaluru, India, with Next-Generation Sequencing Illumina genome sequencing. The quality of the reads obtained from sequencing was verified using FastQC. Raw reads were assembled using SPADES in CPT-GALAXY (https://cpt.tamu.edu), which is an online platform consisting of various software used in genomics. The final single contig obtained was of length 61,010 base pairs (bp). The genome was annotated using PECAAN (https://discover.kbrinsgd.org) software, and final files were generated by DNAmaster software (Pope and Jacobs-Sera, 2018). The annotated genes were verified using National Center for Biotechnology Information (NCBI), HHPRED, and PhagesDB database in PECAAN software. The genome sequence was submitted to NCBI database with an accession number of ON687735.

### Optimum multiplicity of infection and one-step growth curve

*M. smegmatis* culture in the exponential growth phase (OD_600 = _0.4) was serially diluted with LBG media. The initial Colony forming unit (CFU) of the host culture taken was ~5 × 10^7^. A constant PFU of Phage KVT1 was added to tubes containing 500 µl of serially diluted *M. smegmatis* cells in LBG media to form a ratio of 10, 1, 0.1, 0.01, and 0.001 and incubated at 37°C in a rotary shaker at a rate of 150 rpm for 12 h. Culture from each tube was centrifuged at 4,000 g and filtered, and supernatant was collected. PFU of the supernatants were determined. The ratio at which the maximum number of plaques was obtained was considered as the optimum multiplicity of infection (MOI).

One-step growth curve is used to establish the life cycle of bacteriophage. From the growth curve, latency period and burst size of phage can be calculated. One-step growth curve for phage KVT1 was constructed as outlined by Ellis and Delbruck (1939) with minor modifications (Ellis and Delbruck, 1939). Briefly, mid-log phase–grown *M. smegmatis* cells were mixed with phage KVT1 at a MOI of 0.1 and incubated at 37°C in a rotary shaker for 30 min followed by centrifugation and removal of supernatant to eliminate the free/unattached phages. The cell pellet was resuspended in 10.0 ml of LBG media followed by incubation at 37°C and PFU determination at every 30-min interval up to 180 min. Statistical analysis to obtain mean ± S.D. was performed by GraphPad Prism software (version 5.01).

### Thermal pH and stability

Phage stability was assessed at various temperatures (4°C, 15°C, 25°C, 37°C, 45°C, 55°C, and 65°C). Phage KVT1 (1.0 ml) of titer ~10^8^ PFU/ml was incubated for 1 h at each of the various temperatures. After incubation, the phage titer was calculated using the double-layer agar plate method (Capra et al., 2004).

For pH stability test, 100 µl of phage KVT1 (~10^8^ PFU/mL) in phage buffer was diluted in 900 µl of physiological saline with varying pH ranges (1.0–12.0) and incubated at 27°C for 1 h to ascertain the effect of pH on phage stability (Capra et al., 2006). The double-layer agar plate technique was used to measure the phage titer, and the experiments were carried out in triplicates. Statistical analysis to obtain mean ± S.D. was performed by GraphPad Prism software (version 5.01).

### Fluorescence/vonfocal microscopy

Fluorescence microscopy was performed to visualize the interaction of phage KVT1 with its host. The *M. smegmatis* cells grown to mid-log phase (OD_600 = _0.4) were mixed with phage KVT1 at a MOI of 0.5 and incubated at 37°C for different time points. Prior to this step, agarose pads were prepared using 1% low melting agarose on flat glass slides and incubated at 4°C. Phage–host interaction was imaged at 30-min intervals of 0, 30, 60, 90, and 120 min. Two fluorescent stains, 4',6-diamidino-2-phenylindole (DAPI) (25 µg/ml) and Nile Red (20 µg/ml), were used at 0-min time point to stain *M. smegmatis* nucleus and cell membrane, respectively. The host–KVT1 mixture was incubated for 5 min and mounted onto the agarose pad, and the images were captured (Thammatinna et al., 2020). Images were acquired under a Carl Zeiss LSM-780 confocal microscope.

## Results

### Isolation of mycobacteriophage Kashi-VT1

**Figure 1:**
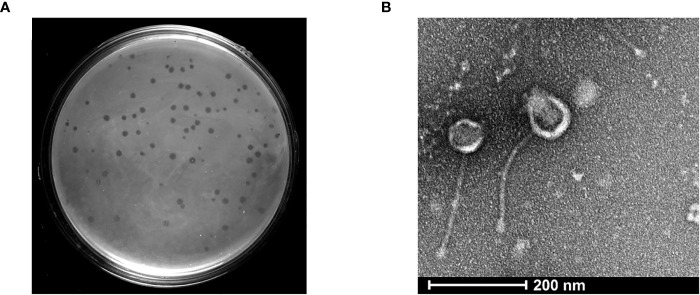
Isolation and structural characterization of mycobacteriophage KVT1 by TEM **(A)** KVT1 plaques on *M. smegmatis* mc^2^ 155 lawn after incubation for 24 h at 37°C. The size of plaques was found to be approximately 2 ± 0.25 mm in diameter. **(B)** TEM image depicting the structure of phage KVT1stained with 2% phosphotungstic acid. Phage KVT1, 273.4 ± 11.2 nm long; head, 67.2 ± 9.1 nm; tail, 206.3 ± 12.4 nm.

To isolate a bacteriophage against *Mycobacterium* species, we selected the non-pathogenic laboratory strain *M. smegmatis* as the host organism. The moist soil sample was collected from a site near the Vishwanath temple located inside the Banaras Hindu University campus, Varanasi, with the geographical co-ordinates as 25.26574′N 82.98791′E. The mycobacteriophage was named as Kashi_VT1 (KVT1). In the particular lot of the samples in which we isolated KVT1, we collected 48 samples and got six phages using *M. smegmatis* as host (12.5% frequency of positives). However, because KVT1 belongs to the cluster K of the mycobacteriophages, which are mostly specific for *M. tuberculosis* also, we decided to characterize and report it first. The clear plaques that were produced by KVT1 on host *M. smegmatis* lawn were measured after 24 h of incubation at 37°C. The average size of the plaques was found to be 2 ± 0.25 mm as depicted in (**Figure 1A**).

### Host range determination

Mycobacteriophage KVT1 was spotted onto *M. smegmatis* and *M. fortuitum* lawns. Clear zones were visible on both the bacterial lawns after 24 h of incubation. The zone was clearer on *M. smegmatis* lawn as compared to *M. fortuitum*. For the determination of host range, a combination of Gram-negative and Gram-positive bacterial genus like *Escherichia coli* (DH5α and BL21 strains), *Pseudomonas aeruginosa*, *Enterococcus faecalis*, *Staphylococcus aureus*, *and Klebsiella pneumoniae* was selected. There was no plaque/zone formation on the lawn of these bacterial hosts.

### Phage morphology

Electron microscopy images of phage KVT1 negatively stained with PTA depicted that the phage features an icosahedral head of size 63.30 ± 3.04 nm, with a non-contractile tail of size 224.62 ± 5.66 nm. The overall length of phage KVT1 is 286.62 ± 1.84 nm. As a result, KVT1 is categorized in accordance with the norms of the International Committee on Taxonomy of Viruses as a member belonging to the family Siphoviridae (**Figure 1B**).

### Restriction digestion of KVT1 genomic DNA

**Figure 2:**
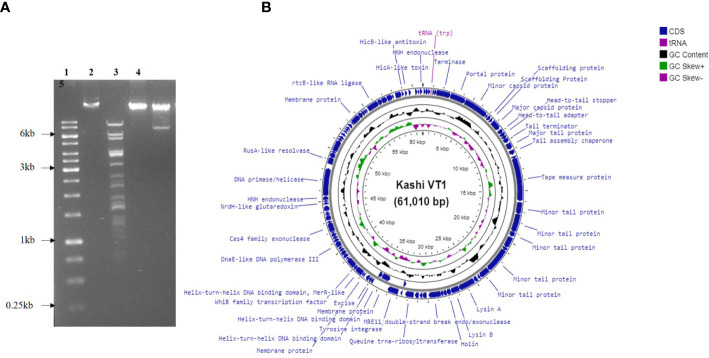
Genomic DNA isolation, restriction digestion analysis, and genome map of KVT1. **(A)** Genomic DNA of phage KVT1 was subjected to restriction digestion by three restriction enzymes, namely, Pst1, Hind III, and Bam H1. Lane 1, DNA marker; lane 2, genomic DNA undigested; lane 3, Pst I; lane 4, Hind III; lane 5, Bam HI. **(B)** Complete genome map of KVT1 consists of 61,010 base pairs. KVT1 dsDNA, visualized by Proksee GC viewer tool (http://stothard.afns.ualberta.ca/cgview_server/). ORFs encoding all genes, annotated genes, tRNA, and total GC content are depicted in sky blue, red, violet, and black colors, respectively.

KVT1 genomic DNA that was isolated using PCI-SDS method was further tested to examine the restriction digestion pattern of the genomic DNA. Phage KVT1 genome when digested with *Pst*1 yielded 16 bands on the agarose gel. There are 42 *Pst*1 sites in the KVT1genome, but, because of the adjacent locations of some of these restriction sites in the genome, very small fragments are generated, which are not visible on the agarose gel. Digestion with *Bam* HI produced a ~7.5-kB band from the main genomic DNA, which indicates the presence of two *Bam* HI restriction sites inside KVT1 genome that also coincides with the theoretical calculations from the sequenced genome. Digestion with *Hind* III resulted in no fallout band; hence, no Hind III restriction sites are present inside KVT1 genome sequence (**Figure 2A**).

### Genome features and annotation of phage KVT1

The complete genome of mycobacteriophage KVT1 is a dsDNA consisting of 61,010 bp with a GC content of 66.5%. The mostly closely related phage to KVT1 is mycobacteriophage CrimD with 92.90% homology, as shown by BLASTn. This places KVT1 in cluster K, subcluster K1 of mycobacteriophages, as shown in the PhagesDB database. Gene prediction by DNA master revealed 101 putative open reading frames (ORFs) and one Transfer RNA (tRNA) gene. All but three of the predicted ORFs are encoded on the forward strand. Forty-one ORFs were assigned a function on the basis of protein homology as depicted in **Table 1**. One tRNA was predicted to specify amino acid tryptophan (anticodon CCA) (**Figure 2B**).

**Table 1:** List of annotated genes with predicted functions in the genome of mycobacteriophage KVT1.

ORF	Predicted Function	Direction	Start	Stop	Length
ORF 6	Terminase, large subunit	+	1,372	2,796	1,425
ORF 7	Portal protein	+	2,808	4,361	1,554
ORF 8	Minor capsid protein	+	4,366	6,840	2,475
ORF 10	Scaffolding protein	+	7,056	7,367	312
ORF11	Scaffolding protein	+	7,408	7,644	237
ORF 12	Major capsid protein	+	7,719	8,654	936
ORF 13	Head-to-tail adaptor	+	8,766	9,152	387
ORF 14	Head-to-tail stopper	+	9,149	9,505	357
ORF 16	Tail terminator	+	9,764	10,189	426
ORF 17	Major tail protein	+	10,290	10,904	615
ORF 18	Tail terminator	+	11,018	11,458	441
ORF 20	Tape measure protein	+	11,868	16,001	4,134
ORF 21	Minor tail protein	+	16,102	17,238	1,137
ORF 22	Minor tail protein	+	17,239	19,005	1,767
ORF 23	Minor tail protein	+	19,005	19,478	474
ORF 26	Minor tail protein	+	20,960	23,392	2,433
ORF 27	Minor tail protein	+	23,404	24,438	1,035
ORF 30	Lysin A	+	25,170	26,825	1,656
ORF 31	Lysin B	+	26,822	27,694	873
ORF 32	Holin	+	27,705	28,145	441
ORF 35	MRE11 double-strand break endo/exonuclease	+	28,739	29,947	1,209
ORF 39	Queuine tRNA-ribosyltransferase	+	31,427	32,281	855
ORF 42	Tyrosine integrase	+	33,003	34,091	1,089
ORF 43	Membrane protein	+	35,097	34,195	903
ORF 44	Immunity repressor	+	35,501	35,124	378
ORF 45	Helix-turn-helix DNA binding domain	+	35,680	35,919	240
ORF 46	Excise	+	35,916	36,182	267
ORF 48	Membrane protein	+	36,701	36,868	168
ORF 50	Helix-turn-helix DNA binding domain, MerR-like	+	37,023	37,814	792
ORF 51	WhiB family transcription factor	+	37,811	38,074	264
ORF 58	DnaE-like DNA polymerase III	+	40,466	41,020	555
ORF 63	Cas4 family exonuclease	+	42,063	42,950	888
ORF 69	NrdH-like glutaredoxin	+	44,711	44,953	243
ORF 71	HNH endonuclease	+	45,156	45,527	372
ORF 72	DNA primase/helicase	+	45,569	48,187	2,619
ORF 73	RusA-like resolvase (endonuclease)	+	48,599	49,297	699
ORF 85	Membrane protein	+	53,212	54,114	903
ORF 90	rtcB-like RNA ligase	+	55,217	56,404	1,188
ORF 96	HicB-like antitoxin	+	58,903	59,100	198
ORF 97	HicA-like toxin	+	59,222	59,437	216
ORF 101	HNH endonuclease	+	60,726	60,953	228

Open reading frames (ORFs) with the predicted functions were determined from NCBI database. +The direction of the open reading frame (ORF) in the genome is + or forward orientation (conventionally from leftwards to rightwards direction).

Genome sequence of KVT1 contains the following gene groups (**Figure 2B**; **Table 1**):

a) DNA packaging genes include terminase (gp6) and portal protein (gp7). Terminase acts both as an ATP motor and as an endonuclease for the translocation of phage DNA into empty head and cutting the phage DNA to initiate proper packaging (Fujisawa and Morita, 1997). The portal protein acts as a channel for two ways transit of phage DNA and, in case of tailed bacteriophages, acts as an attachment point for tail machinery (Leiman and Shneider, 2012).
b) Structural genes include minor capsid protein (gp8), scaffolding protein (gp10 and gp11), major capsid protein (gp12), head-to-tail adaptor (gp13), head-to-tail stopper (gp14), tail terminator (gp16), major tail protein (gp17), tail assembly chaperone (gp18), tape measure protein (gp20), and minor tail protein (gp21, gp22, gp23, gp26, and gp27).
c) Virion release genes includes lysin A (gp30), lysin B (gp31), and holin (gp32), which take part in host cell lysis during the lytic cycle.
d) Lysogeny genes include tyrosine integrase (gp30) and excise (gp46).
e) Replication and genome maintenance genes include MRE 11 double-strand break endo/exonuclease (gp35), queuine tRNA-ribosyltransferase (gp39), helix-turn-helix DNA binding domain, MerR-like (gp39), WhiB family transcription factor (gp51), DnaE-like DNA polymerase III (gp58), Cas4 family exonuclease (gp63), NrdH-like glutaredoxin (gp69), HNH endonuclease (gp71), DNA primase/helicase (gp72), RusA-like resolvase (endonuclease) (gp73), and rtc-B like RNA ligase (gp90).

In the genome circular map, DNA packaging genes, structural genes, virion release genes, and lysogeny genes are clustered together, whereas replication and genome maintenance genes are clustered together at another locus.

### Optimum multiplicity of infection and one-step growth curve

**Figure 3:**
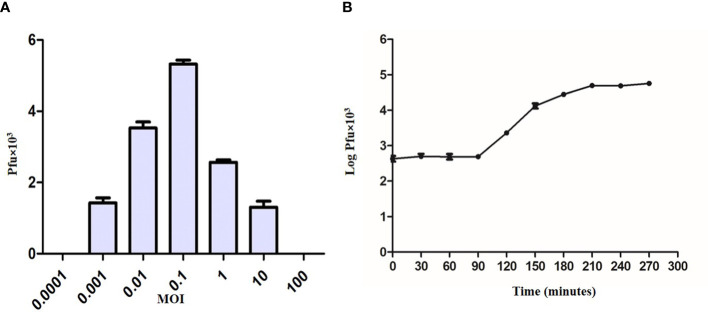
Multiplicity of infection and one-step growth assay of bacteriophage KVT1. **(A)** Determination of multiplicity of infection (MOI) of KVT1. The X-axis represents MOI, whereas the Y-axis represents the PFU of the phage KVT1. Optimum MOI for the phage KVT1 was determined to be 0.1. **(B)** One-step growth curve assay of the bacteriophage KVT1. The X-axis represents time in minutes, whereas the Y-axis represents the log PFU (× 10^3^) of the phage KVT1. The latent period and the burst size of phage KVT1 were determined to be 90 min and 102, respectively. The plateau phase of the growth reached after 180 min of the infection.

The ratio of adsorbed infecting agents to susceptible host targets is referred to as the MOI. The optimum MOI for KVT1 was found to be 0.1 for *M. smegmatis*, which indicates that the presence of one phage per 10 bacteria induces maximum progeny phages in the culture medium (**Figure 3A**).

To understand the life cycle of a KVT1 on host *M. smegmatis*, one-step growth curve assay was performed. Log of the PFU/ml was plotted against time to obtain one-step growth curve. One-step growth curve constitutes three different phases: latent period, burst or rise period, and plateau period (Delbrück, 1940).

The latent period refers to the time period before the release of phage particles into the culture medium (Wang et al., 1996). Because no new phage particles are released during this period, the plaque count stays almost constant. The latent period of phage KVT1 was determined to be 90 min. The burst size refers to the average production of infectious virus per cell. It is calculated as PFU/ml during the plateau period and PFU/ml during the latent period. Following the analysis of the experimental data, the burst size of phage KVT1 was determined to be 102. During the plateau stage due to the lysis of almost all the host cells by the phage infection, there is no further rise in the PFU of the phage. For KVT1, the plateau phase of the growth reached after 180 min of the infection (**Figure 3B**).

### Thermal and pH stability

**Figure 4:**
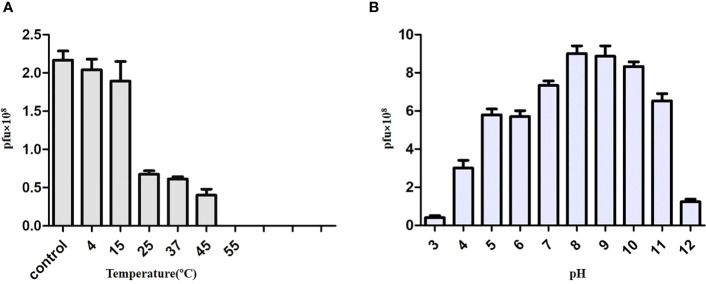
Effect of temperature and pH on the stability and viability of the phage KVT1. **(A)** Effect of temperature on the phage KVT1. The X-axis represents temperature in degree centigrade, whereas the Y-axis represents PFU (× 10^7^) of KVT1. Maximum stability of the phage KVT1 was determined in the temperature range of 4°C–15°C, whereas the phage viability was up to 45°C. **(B)** Effect of pH on the phage KVT1. The X-axis represents pH, whereas the Y-axis represents PFU (× 10^7^) of KVT1. The maximum stability of the phage KVT1 was determined in the pH range of 8 to 10, intermediate stability was in the pH range of 4 to 7 (acidic range) and at pH 11 (alkaline range), whereas the phage viability was minimum at pH 3 and 12.

The temperature and pH stabilities of phage KVT1 were assessed by calculating the variations in PFU in response to the changes in temperature and pH, respectively. The thermal stability of KVT1 was examined for 1 h at temperature 4°C to 65°C. The results demonstrated that, at 4°C, the phage KVT1 retained almost 100% infectivity, and there was no significant change in the stability of phage preparation. After incubation of KVT1 at 15°C, the PFU of phage KVT1 reduced to 61%, whereas, at 25°C and 37°C, the number of viable phages (PFU) reduced to 26% and 24%, respectively, as compared to that of the control. At 45°C, only 15% viable phages remained, and, at 55°C, the viability of KVT1 was completely lost. Thus, the maximum stability of the phage KVT1 was determined in the temperature range of 4°C–15°C, whereas the phage viability was up to 45°C (**Figure 4A**).

The pH stabilities of KVT1 were tested across a pH range of 3 to 12. The pH stability experimental findings depicted that the mycobacteriophage KVT1 had broad pH stability at the above range. It was observed that phage KVT1 retained maximum viability at pH 8, 9, and 10. At pH 5 and 6, the viability of KVT1 was 39% less than that of pH 8, and, at pH 7, the viability of KVT1 reduced to 19%. In the extreme acidic pH range of 3 and 4, the viability of phage KVT1 was reduced by 67% and 96%, respectively, as compared to that of pH 8, and, at the extreme basic pH of 11 and 12, the viability of KVT1 was 28% and 87% less than that of pH 8. Thus, the maximum stability of the phage KVT1 was determined in the pH range of 8 to 10, intermediate stability was in the pH range of 4 to 7 (acidic range) and at pH 11 (alkaline range), whereas the phage viability was minimum at pH 3 and 12 (**Figure 4B**).

### Fluorescence/confocal microscopy

**Figure 5:**
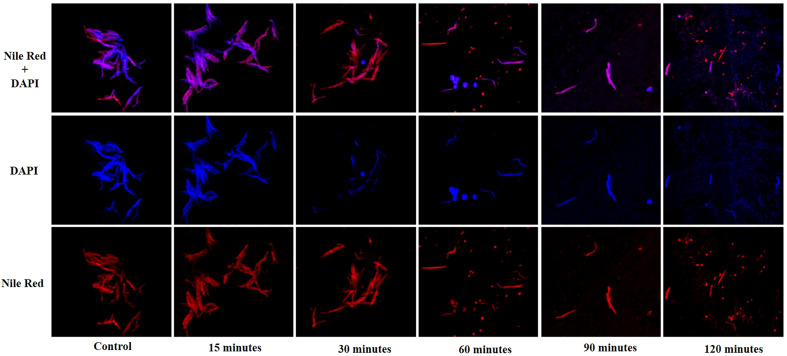
Confocal microscopy images showing lysis of the host *M. smegmatis* mc^2^ 155 cells by mycobacteriophage KVT1. *M. smegmatis* cells were grown to mid-log phase in liquid culture and infected by phage KVT1 at a MOI of 5 and incubated at 37°C. For fluorescence imaging, cell membrane (red) and nucleoid region (blue) were stained by Nile Red and DAPI, respectively. Stained cells (10 µl) were placed onto an agar pad at 15-, 30-, 60-, 90-, and 120-min intervals *M. smegmatis* cells before infection were taken as control. Blue-colored nuclear blobs start to appear at 30 min that increases appreciably at 60 min, indicating cell death. Maximum nuclear and cell debris are visible at 120 min after incubation.

The lysis of host *M. smegmatis* by phage KVT1 was visualized by fluorescent microscopy. *M. smegmatis* cells were grown upto mid-log phase in liquid culture and infected by phage KVT1 at a MOI of 5 and incubated at 37°C. For fluorescence imaging, cell membrane (red) and nucleoid region (blue) were stained by Nile Red and DAPI, respectively, and visualized at a time interval of 15, 30, 60, 90, and 120 min. Around 30 min after the incubation was started, initiation of nuclear blob was visible, and, around 60 min, several nuclear blobs are clearly visible, which indicates cell death. As the time period increases, the lysis of host *M. smegmatis* cells proceeds further, and, at 120 min, the cell wall and nuclear debris are clearly visible, which indicates almost complete lysis of the host cells (**Figure 5**).

## Discussion

Antibiotic therapy to treat tuberculosis possesses major disadvantages like emergence of drug resistance in *M. tuberculosis* bacteria. Thus, alternative therapies to treat tuberculosis are on high demand, and phage therapy among them is highly promising (Chaturvedi et al., 2007). As phages co-evolve with their host, there is least chance of developing host resistance unlike that of antibiotics. Another advantage of phage over antibiotics is that, unlike phages, antibiotics have broad host range posing threats to helpful microbiota residing in the human body (Gondil and Chhibber, 2018).

Owing to the slow growth pattern and pathogenicity of *M. tuberculosis*, the majority of mycobacteriophages have been isolated against laboratory species *M. smegmatis*, which are subsequently tested on *M. tuberculosis* (Koskella and Brockhurst, 2014). Apart from therapeutic purposes, mycobacteriophages can be used for TB diagnostics in addition to serving as model systems for the study of mycobacteria molecular genetics (Lelovic et al., 2020). In the genus *Mycobacterium*, KVT1 is able to target the fast-growing members of the clade/sub-genera *Fortuitum-Vaccae* (new nomenclature: *Mycolicibacterium*). It was also tested against three common gram-negative and two gram-positive bacteria. However, it was not tested against the slow-growing *Mycobacterium* species like *M. tuberculosis* and *M. marinum*. Bacteriophages maintain a high specificity toward their host even at the strain level and do not infect the other species. The host range experiments have been performed with different bacterial hosts like *E. coli*, *Klebsiella pneumoniae*, *Staphylococcus aureus*, *and Enterococcus faecalis* in addition to *Mycobacterium* species, and the plaques were observed only against *Mycobacterium* species, which indicates that specificity to target only the respective host lies at the genomic level.

All bacteriophages possess a receptor-binding protein (RBP) (tail fiber protein/tail spike protein/minor tail protein) that imparts specificity to these phages. These RBPs recognize different sugar moieties, lipids, peptides, etc., present on the outer surface of bacterial cell walls, which are different for both host and phage. In the report of Arutyunov et al. (2014), it was demonstrated that mycobacterial phage L5 minor tail protein gp6 can bind to *Mycobacterium* species but cannot bind to *E. coli*, *Salmonella*, and *Campylobacter* species. L5 genome sequence was compared to other phages like D29, Bxb1, and TM4 phages to know the possible RBP genes among the genes encoding tail proteins. However, the homolog of the gene was found in the genome of phage D29, but not in TM4 and Bxb1, showing the maximum specificity among bacteriophages (Arutyunov et al., 2014).

In addition to this, other bacteriophages only encode a single endolysin that cleaves the bonds of the peptidoglycan layer of the cell wall. However, owing to the complex cell wall composition of *Mycobacterium* sp., mycobacteriophages encode two endolysins, namely, endolysin A that cleaves the bonds in the peptidoglycan and endolysin B that cleaves the ester bond in mycolyl-arabinogalactan to attain efficient killing. Mycobacteriophages also encode a holin protein that creates holes in the plasma membrane of mycobacterial hosts and helps the endolysin to easily target the complex cell wall during cell lysis. KVT1 genome contains tail proteins (gp 21–gp 27), lysin A (gp30), lysin B (gp31), and holin (gp32), which depicts that it is highly evolved and specific to target *Mycobacterium* species but no other bacterial species.

As per Jacob Serra et al., mycobacteriophages from cluster K, cluster G, and cluster A (sub-clusters A2 and A3) can efficiently infect *M. tuberculosis* and thus can be applied for therapeutic purposes (Jacobs-Sera et al., 2012). Phages should possess specific characteristics that include a strict lytic life cycle, absence of toxic genes (antibiotic-resistant genes), broader host range, good transduction, and virulence potential (Gill and Hyman, 2010) for successful applications in therapy. Phages isolated from the natural environment may or may not meet the criteria for successful phage therapy; hence, they might require modifications. These modifications can be achieved by conventional homologous recombination, Bacteriophage Recombineering of Electroporated DNA (BRED), CRISPR-Cas, and CRISPY-BRED (Pires et al., 2016). When examining KVT1 as a prospective agent for phage therapy, this specificity trait can be highly advantageous because phage specificity can allow only the pathogenic/non-pathogenic mycobacterial species to be targeted while protecting the other useful bacterial flora from other genera. Despite the fact that many mycobacteriophages have been isolated against *M. smegmatis*, only a subset of them can infect and kill the pathogenic strain *M. tuberculosis*. Thus, the continuous discovery and characterization of novel mycobacteriophages is highly advisable as it will add to the diversity and repertoire of potential therapeutic phages. Antibiotic-resistant *M. chelonae* that causes severe disseminated cutaneous infections in a patient was treated with a combination of antimicrobials, and surgical and single bacteriophage therapy (Mycobacteriophage Muddy) gave excellent clinical results with stable improvement (Little et al., 2022). A patient with MDR *M. abscessus* infection was treated with a cocktail of three mycobacteriophages Muddy, BPs, and ZoeJ that belonged to clusters AB, G, and K, respectively, and their genetically engineered mutants (temperate to lytic in BPs and ZoeJ) *via* intravenous injections gave substantial resolution of infected skin nodules with no evidence of phage neutralization (Dedrick et al., 2019). Muddy being lytic has been used directly, whereas BPs and ZoeJ, both temperate mycobacteriophages, were engineered and converted into lytic phages, whereas BPs was also engineered for broader host range for therapy. Because cluster K mycobacteriophages are known to infect the pathogenic *M. tuberculosis* strains, the isolated mycobacteriophage KVT1 also being a Cluster K phage possesses a greater probability for binding to pathogenic *M. tuberculosis* strains. In addition, KVT1 being a temperate phage like ZoeJ will need to be engineered into lytic form for further therapeutic utility (after specificity determination against *M. tuberculosis*) or after minor genetic alterations for host range.

BLAST results from PhagesDB database depicts that mycobacteriophages belonging to cluster K are polyvalent and have the ability to infect both *M. tuberculosis* and *M. smegmatis* (Hatfull, 2019; Fan et al., 2015). Hendrix (1999) reported that all the dsDNA tailed phages share common ancestry (Hendrix et al., 2002). However, Moura de Sousa et al. (2021) reported that there is more diversity in temperate phages as compared to virulent ones and especially those which have recombinases, transposases, and non-homologous end joining, indicating that both homologous and illegitimate recombination contribute to the gene flow. Although virulent phages have lower rate of recombination, they also undergo recombination during lysis in the hosts that are having prophages in their genome (Moura de Sousa et al., 2021). KVT1 is a temperate phage that possesses tyrosine integrase (gp30) and excise (gp46) indicating that this phage might be involved in the process of recombination leading to increased genetic diversity. Although the phage family/phyla are prominent barriers for genetic exchange, their lifestyle is not the determining factor. The genes like tyrosine integrase and/or excise can be knocked out or mutated to eliminate the lysogenic property of KVT1 for incorporating it in therapeutic applications (**Table 1**) (Guerrero-Bustamante, et al., 2021).

Before subjecting a phage to therapeutic purposes, it is important to determine its characteristics such as optimum MOI, latent period, burst size, effect of temperature, and pH. KVT1 has a latent time and burst size of 90 min and 102, respectively, which is comparable to the latent time and burst size of mycobacteriophages MS6 and SWU1 belonging to the clusters F1 and A2. The latent periods of MS6 and SWU1 are 120 and 90 min and burst sizes are 147 and 200 phage particles, respectively (Sinha et al., 2020; Jończyk-Matysiak, 2019). Lysis of host cells by KVT1 was depicted through fluorescence microscopy. The images depict starting of nuclear blob formation at 30 to 60 min and maximum lysis at 120 min, which directly correlates with the one-step growth assay that exhibits rise in phage particles after the burst in the medium from 90 to 150 min (with the mid-point of 120 min) (**Figure 5**).

Determination of viability of phages in a certain temperature and pH range also helps to ascertain long term storage conditions for phages. Some research indicates a possible link between phage morphology and their persistence in harsh environments (Ly-Chatain, 2014). In our study, KVT1 was most stable at 4°C retaining 100% viability, and phage activity was completely lost at 55°C, whereas, at the physiological temperature of 37°C, phage activity was reduced almost 3.5 times as compared to 4°C. Other studies also demonstrated that phages remain stable in the temperature range of 45°C–65°C (Jurczak-Kurek et al., 2016; Sellers and Runnals, 1961). Phage stability is greatly influenced by acidic and alkaline pH. KVT1 was comparatively stable in the pH range of 7 to 10 that also includes the physiological pH 7.4. In other studies, including mycobacteriophages and other phages, a similar trend was followed (Bartek et al., 2016; Kim et al., 2019; Montso et al., 2019).

Antimicrobial drug resistance has emerged as a worldwide public health concern posing threats to the effective prevention and management of infectious diseases. Most, if not all, antibiotics are no longer effective due to the emergence of MDR microorganisms (Lu and Koeris, 2011). In case of *Mtb*, XDR and completely drug-resistant strains are difficult to control. Thus, effective tools to control and combat *M. tuberculosis* and its multidrug resistance strains are urgently needed (Rohde et al., 2018). Mycobacteriophages have been isolated and genomically described in vast numbers, offering insights into viral diversity and evolution as well as stimulating the need for the development of mycobacterial genomic techniques to treat mycobacterial infections. Because most mycobacteriophages belonging to cluster K are specific against *M. tuberculosis*, for example, ZoeJ (Pope et al., 2011), KVT1 that also belongs to cluster K holds potential as a promising anti-mycobacterial candidate. Challenges are ethical issues, lytic-lysogeny conversion, preparation of host-free phage sample, etc., but, after addressing these issues, phage therapy can be successfully implemented with positive patient outcomes that are resistant to multiple classes of antibiotics.

## Data availability statement

The datasets presented in this study can be found in online repositories. The names of the repository/repositories and accession number(s) can be found in the article/supplementary material.

## Author contributions

TN isolated, characterized the mycobacteriophage KVT-1 and wrote the manuscript. AK, RS, and LJ have contributed in the isolation and characterization of mycobacteriophages. AS has helped in the confocal microscopy. LT helped in the genome assembly/ annotation and in writing of the manuscript. AG has conceptualized and supervised the isolation/ characterization of the mycobacteriophage and helped in preparing the manuscript. All authors contributed to the article and approved the submitted version.
